# Is Saglin a mosquito salivary gland receptor for *Plasmodium falciparum*?

**DOI:** 10.1186/s12936-018-2634-5

**Published:** 2019-01-03

**Authors:** David A. O’Brochta, Robert Alford, Robert Harrell, Channa Aluvihare, Abraham G. Eappen, Tao Li, Sumana Chakravarty, B. Kim Lee Sim, Stephen L. Hoffman, Peter F. Billingsley

**Affiliations:** 1 Department of Entomology and The Institute for Bioscience and Biotechnology Research, University of Maryland, College Park, 9600 Gudelsky Drive, Rockville, MD 20850 USA; 2 Present Address: Foundation for the National Institutes of Health, 11400 Rockville Pike, Suite 600, North Bethesda, MD 20852 USA; 3 University of Maryland Insect Transformation Facility, The Institute for Bioscience and Biotechnology Research, 9600 Gudelsky Drive, Rockville, MD 20850 USA; 4 Sanaria Inc., 9800 Medical Center Drive, Suite A209, Rockville, MD 20850 USA

**Keywords:** *Plasmodium falciparum*, Sporozoite, Mosquito, Salivary gland, *Anopheles stephensi*, Saglin, Receptor, *Anopheles gambiae*

## Abstract

**Background:**

Saglin, a 100 kDa protein composed of two 50 kDa homodimers, is present in the salivary glands of *Anopheles gambiae* and has been considered an essential receptor for sporozoites (SPZ) of *Plasmodium berghei* and *Plasmodium falciparum* (Pf), allowing SPZ to recognize, bind to, and infect mosquito salivary glands. Spatial and temporal patterns of Saglin expression reported here, however, suggest that this model does not fully describe the Saglin–SPZ interaction.

**Results:**

Saglin protein was detected by indirect immunofluorescence microscopy only in the medial and proximal-lateral lobes, but not in the distal-lateral lobes, of the salivary glands of *An. gambiae*; the pattern of expression was independent of mosquito age or physiological state. These results were confirmed by steady-state Saglin transcript and protein expression using qRT-PCR and Western-blot analysis, respectively. Saglin was localized to the basal surface of the cells of the medial lobes and was undetectable elsewhere (intracellularly, on the lateral or apical membranes, the cells’ secretory vacuoles, or in the salivary duct). In the cells of the proximal lateral lobes of the salivary glands, Saglin was distinctly intracellular and was not localized to any of the cell surfaces. Transgenic *Anopheles stephensi* were produced that expressed *An. gambiae* Saglin in the distal lateral lobes of the salivary gland. Additional Saglin expression did not enhance infection by PfSPZ compared to non-transgenic siblings fed on the same gametocyte-containing blood meal.

**Conclusions:**

The absence of Saglin in the distal lateral lobes of the salivary glands, a primary destination for SPZ, suggests Saglin is not an essential receptor for *Plasmodium* SPZ. The lack of any correlation between increased Saglin expression in the distal lateral lobes of the salivary glands of transgenic *An. stephensi* and PfSPZ infection is also consistent with Saglin not being an essential salivary gland receptor for *Plasmodium* SPZ.

## Background

During the course of infection of malaria-susceptible *Anopheles* mosquitoes, *Plasmodium* parasites must traverse two insect single cell-layered epithelia before being transmitted by mosquitoes that subsequently feed on susceptible hosts [1, 2]. The last insect epithelial barrier to *Plasmodium* transmission is that of the salivary gland. After being released into the haemolymph from oocysts attached to the basal surface of the midgut epithelium, sporozoites (SPZ) must migrate through the haemocoel to the basal lamina of the salivary glands where they attach, invade, traverse and finally emerge from the salivary gland cells into the apical secretory cavity of the infected cells and then into the salivary duct [3]. The parasites’ interactions with epithelial cells are critically important since the midgut and salivary glands are the only insect tissues to be invaded by *Plasmodium*. There is great interest in understanding the molecular bases of these interactions because they might become targets for molecular therapeutic agonists and antagonists.

A better understanding and targeted manipulation of mosquito-*Plasmodium* interactions could be important for optimizing development and manufacture of whole *P. falciparum* (Pf) SPZ vaccines and infectious PfSPZ used for controlled human malaria infections (CHMI). PfSPZ raised in aseptically reared *Anopheles stephensi* are used to manufacture a family of Sanaria^®^ PfSPZ products. These include PfSPZ Vaccine (radiation attenuated PfSPZ) [4–9], PfSPZ Challenge (infectious PfSPZ) [10–20], and PfSPZ-GA1 (genetically attenuated PfSPZ) [21]. PfSPZ Challenge is also used with anti-malarial drugs in PfSPZ-CVac (chemo-attenuated PfSPZ) [22–24]. The efficiency of production of PfSPZ for these products is directly related to the PfSPZ infection intensities and prevalence rates of the aseptic mosquitoes [25, 26]. Thus, identifying the molecular physiological mechanisms that can be manipulated to improve mosquito infection rates by PfSPZ is important for optimizing the efficiency of production of PfSPZ-based products.

Interspecific transfer of salivary glands into *Plasmodium*-infected mosquitoes has demonstrated that SPZ-salivary gland interactions are species-restricted [27]. Furthermore, anti-salivary gland antibodies and certain lectins are capable of interfering with SPZ invasion of salivary glands [28–31], and proteins recognized by these antibodies may play either a direct or indirect role in the invasion process. Saglin, a salivary gland protein of *Anopheles gambiae* with no homolog in *An. stephensi,* is recognized by monoclonal antibody 2A3 (mAb2A3). Despite its species specificity, Saglin has been proposed to play a particularly important role in salivary gland-SPZ interactions and in salivary gland invasion [30, 32, 33].

In their initial characterization, Brennan et al. [30] reported that mAb2A3 bound exclusively to the medial and lateral lobes of the salivary glands of female *An. gambiae*. Their immuno-electron microscopy localization study showed sparse localization dispersed throughout the salivary gland and their light microscopy study using indirect immunolocalization methods also showed a diffused distribution of Saglin over all major morphological regions of the salivary gland. Saglin was expressed in *An. gambiae* only after the adult mosquitoes were 6 days old and was not among the proteins secreted by the salivary glands [30]. Interestingly, female mosquitoes fed mAb2A3 10 days after the infectious blood meal harbored 73% fewer *Plasmodium yoelii* (Py) SPZ in their salivary glands [30]. They reasoned at the time that because the antibody had not affected the prevalence of infection but only the intensity of PySPZ infection in the salivary glands, that the antibody reduced the available number of target sites with which PySPZ interacted during the initial stages of salivary gland invasion.

Saglin is a 100 kDa protein consisting of a homodimer of 50 kDa subunits [33]. Partial determination of the Saglin amino acid sequence allowed the identification of the *An. gambiae Saglin* gene within the published genome sequence of this mosquito [34]. An analysis of the resultant amino acid sequence did not reveal any transmembrane domains, but a putative signal sequence was identified. Unlike Brennan et al. [30], Okulate et al. reported Saglin in the saliva of female *An. gambiae* [30, 33] and also suggested that Saglin might be a molecular cue for free SPZ within the haemocoel that facilitates their localization to the distal regions of the salivary glands.

Gosh et al. [32] reported Saglin binding to *Plasmodium* thrombospondin-related anonymous protein (TRAP also known as sporozoite surface protein 2 [35]) and concluded that Saglin was a receptor for both PfSPZ and *Plasmodium berghei* (Pb) SPZ. This hypothesis is hereby referred to as the “Saglin model” of *Plasmodium* SPZ infection. Injection of mAb2A3 as well as Saglin double-stranded RNA (dsRNA) into the haemocoel of oocyst infected *An. gambiae* reduced the intensity of PfSPZ and PbSPZ infections in the salivary glands, lending support to the Saglin model [32].

In the present study, the Saglin model was assessed by examining the temporal and spatial patterns of *Saglin* gene expression, and by determining the effects of *Saglin* over-expression on infection intensity. *Plasmodium* SPZ invade the distal regions of the medial and lateral lobes of the salivary glands of infected female mosquitoes [36] and if the Saglin model is accurate protein distribution should be consistent with known patterns of SPZ infection. This is a strong test of the model since patterns of *Saglin* expression and *Plasmodium* infection inconsistent with one another will not be compatible with the current model. The model also proposes a direct interaction between Saglin and TRAP; decreased Saglin expression should thus correlate with reduced infection intensity. Thus, the relationship between Saglin expression and infection intensity is explored in *An. stephensi* when *An. gambiae* Saglin is over-expressed.

The present results fail to support the proposed Saglin model. Using indirect immunofluorescence microscopy and laser scanning confocal microscopy, Saglin expression was observed only in or on the medial- and proximal-lateral lobes of the salivary glands of *An. gambiae.* Saglin was undetectable in the distal lateral lobes, a major site of *Plasmodium* invasion and was not present in the salivary glands of *An. stephensi*, a highly susceptible malaria vector. These data were supported by protein localization using Western blot and steady-state transcript-abundance using quantitative real time polymerase chain reaction (qRT-PCR). Transgenic *An. stephensi* expressing *An. gambiae* Saglin specifically in the distal lateral lobes of the salivary glands failed to show any increased intensity or prevalence of *P. falciparum* oocyst or PfSPZ infections.

## Methods

### Mosquitoes

*Anopheles gambiae* G3 and *An. stephensi* SDA 500 were used in these studies and were reared as adults at 28 °C, 80% humidity. Larvae were fed ground TetraMin^®^ Tropical Flakes ad libitum. Adults were fed 10% sucrose solution and adult females fed on mice to support mosquito colony maintenance.

### Mosquito transformation

Mosquito transformation was performed in the University of Maryland’s Insect Transformation Facility (http://ibbr.umd.edu/insect_transformation) using standard protocols [37, 38]. A *piggyBac* vector with a *3xP3DsRed* marker gene and a transgene consisting of the promoter from the *anopheline antiplatelet protein* (*AAPP*) gene from *An. stephensi* regulating the expression of the *Saglin* open reading frame and the 3′ untranslated region (3′UTR) of the SV40 virus was constructed and introduced into the genome of *An. stephensi* [39, 40]. *AAPP* is expressed specifically in the distal lateral lobes of the salivary glands of female *An. stephensi* [40]. The region 5′ of the open reading frame of *AAPP* (nucleotides 4–1687; accession number A212871) which includes the promoter was isolated from *An. stephensi* using PCR and was used to ensure that *Saglin* is expressed specifically in the distal lateral lobes of the salivary glands of female mosquitoes. A 1541 bp fragment of DNA was synthesized consisting of 1239 bp (412 aa) of the *Saglin* open reading frame (nucleotides 1–1239; AY846632) and 302 bp containing the 3′ UTR of SV40 (GenScript Corporation, Piscataway, NJ). The promoter and transcription unit were assembled in pSL1180fa and then transferred as single fragment into the *piggyBac* 3xP3DsRed vector [41].

### Inverse PCR

The presence and location of integrated gene vectors in transgenic mosquitoes was determined by inverse PCR [42]. Genomic DNA from the equivalent of one mosquito was digested to completion with restriction endonuclease *Msp*I or *Hae*III according to the manufacturer’s recommendation (New England Biolabs) in a reaction volume of 50 μL. On completion of the digestion step, the restriction endonuclease was heat inactivated. The digested genomic DNA was then ligated under dilute conditions to bias the formation of monomeric circular products. The final ligation reaction was 300 μL and contained all the digested DNA from the 50 μL restriction digest and 40 units of ligase (New England Biolabs). Ligation was performed overnight at 16 °C. The DNA in the ligation reaction was precipitated, washed and resuspended in 15 μL of sterile distilled water. One half of the DNA was then used as a template in two identical PCR reactions except for the presence of different primers. To amplify the 5′ terminus of the integrated *piggyBac* element and flanking genomic DNA the primers used were 5′-TCT TGA CCT TGC CAC AGA GG-3′ and 5′-TGA CAC TTA CCG CAT TGA CA-3′. To amplify the 3′ terminus of the integrated *piggyBac* element and flanking genomic DNA the primers used were 5′-AAA CCT CGA TAT ACA GAC CGA TAA AAC AC-3′ and 5′-CAT TTG CCT TTC GCC TTA TTT TAG A-3′. The PCR cycle conditions were: one cycle at 95 °C for 3 min, followed by 30 cycles of 95 °C for 30 s, 52 °C for 30 s, 72 °C for 2 min and finally one cycle at 72 °C for 5 min. The DNA sequences of the PCR products were determined.

### *Plasmodium falciparum* culture and mosquito feeding

*Plasmodium falciparum* strain NF54 cultures were initiated from vials of cryopreserved parasites and maintained at 5% haematocrit in type O+ human red blood cells (RBCs) and complete growth medium (RPMI-1640 supplemented with type O+ human serum and hypoxanthine) with passage of cultures every 3–4 days [25, 43]. Gametocytes were induced by maintaining the cultures with daily complete growth medium replacement but without the addition of uninfected erythrocytes for 14–18 days. Cultures were selected for feeding at 14–18 days post gametocyte induction based upon the proportion of stage V gametocytes present in the culture. Stage V gametocytes were combined with uninfected type O+ human erythrocytes and human O+ serum and fed to mosquitoes via an artificial membrane feeder. Mosquitoes were then maintained at 25 ± 2 °C and 70–80% relative humidity on water and solid sugar until dissection to estimate *P. falciparum* infection rates at either the oocyst (days 7–9 post feeding) or PfSPZ (days 16–18 post feeding) stage.

### *Anopheles* infection with *Plasmodium falciparum*

Transgenic *An. stephensi* with the *AAPP*-*Saglin* transgene (*As*-*AAPPSaglin*) were outcrossed to wild type *An. stephensi* and transgenic heterozygous progeny were selected and separated according to sex. These heterozygotes were again outcrossed to wild type *An. stephensi* and female heterozygous transgenic and wild type progeny were infected with *P. falciparum*. Approximately 300 female mosquitoes (~ 150 heterozygous transgenic, ~ 150 non-transgenic) were placed in each of three cages and fed *P. falciparum* gametocytes as described above. After 7–9 days at 25 ± 2 °C, approximately 150 mosquitoes were removed from each cage, separated into those expressing DsRed (transgenic) and those without DsRed expression (non-transgenic) and then dissected to remove the midguts. The midguts were then examined by microscopy for the presence of oocysts and the numbers recorded. At 16–18 days post-feeding the remaining mosquitoes in the three cages were separated based on their genotypes (transgenic vs non-transgenic), salivary glands were isolated and the numbers of PfSPZ in each were counted using a hemocytometer.

### Indirect immunofluorescence

Salivary glands were dissected in phosphate buffered saline (PBS; 137 mM NaCl, 2.7 mM KCl, 10 mM Na_2_HPO_4_, 2 mM KH_2_PO_4_, pH 7.4), and fixed in 4% paraformaldehyde in PBS for 15 min at ambient temperature. Fixative was removed and the tissues washed for 3 × 5 min in methanol. Methanol was removed and replaced with 200 μL of a 1:10 solution of H_2_O_2_ and methanol for 15 min at room temperature, then the tissues were washed 3 × 15 min in a 1:1 mixture of methanol and PBS with 0.1% TritonX100 (PBST). The tissues were washed in PBST with 1% bovine serum albumin (PBSBT) 5 × 5 min and then blocked in PBSBT for 1 h at ambient temperature. The tissues were again washed in PBST 3 × 5 min at which point the *An. gambiae* Saglin-specific mAb2A3, diluted 1:200 in PBST, was added to the tissue and incubated overnight at 4 °C [30]. The mAb2A3-containing solution was removed and the tissues were washed with PBSBT 3 × 5 min followed by 3 × 15 min washes. Alexa Fluor^®^ 555 goat anti-mouse antibody (A31622, Invitrogen, Eugene, OR) was diluted 1:200 in PBST, added to the tissues and incubated for 2 h at ambient temperature in the dark. The Alexa Fluor^®^ 555 Goat Anti-Mouse antibody-containing solution was removed and the tissues were washed PBSBT for 3 × 5 min followed by 3 × 15 min washes. When required, the first 15-min wash with PBSBT also contained 300 nM DAPI (4′, 6-diamidino-2-phenylindole, 46190, Pierce, Rockford, IL). The processed salivary glands were mounted on glass slides under coverslips in VECTASHIELD^®^ Mounting Medium (Vector Laboratories, Burlingame, CA) and observed with either a Zeiss Axio Imager.A1 under phase contrast or fluorescence illumination using the Rhodamin-shift-free filter set (EX BP 546/12, BS FT 560, EM BP 575–640) or with a Zeiss 710 laser scanning confocal microscope in which the 405 nm and 561 nm lasers were used for fluorochrome excitation. Indirect immunofluorescence experiments was repeated at least four times and each experiment always involved dissecting approximately 10–15 salivary glands from females of non-transgenic *An. stephensi*, transgenic *An. stephensi* and non-transgenic *An. gambiae* and all glands were processed in parallel.

### qRT-PCR

Salivary glands from unfed adult female *An. gambiae* at 4 days post eclosion were dissected in cold PBS. The medial glands were separated from the lateral glands (proximal + distal regions) and placed immediately in TRIzol^®^ Reagent (Invitrogen). Three pools of glands (each with approximately 10–15 medial or lateral lobes) were collected (biological replicates) and RNA was isolated according to the manufacture’s recommendations (Invitrogen). Complementary DNA (cDNA) was synthesized using the SuperScript^®^ III First-Strand Synthesis System for RT-PCR according to the manufacturer’s recommendations (Invitrogen). qRT-PCR was performed using an Applied Biosystems 7300 Real-Time PCR System using Saglin primers SG746F: 5′-CGA CCT TGT CCG GCA GTC CG-3′ and SG1089R: 5′-CTG CCG TGC CGC CTT TAC CA-3′, and ribosomal protein, S7 primers (normalization control); AsS7f: 5′-TGC GGC TTC AGA TCC GAG TTC-3′ and AsS7r 5′-TTC GTT GTG AAC CCA AAT AAA AAT C-3′. All cDNA samples were analysed in triplicate (technical replicates) under the following cycle conditions in the presence of SYBR Green: 95 °C for 1 min followed by 39 cycles consisting of 95 °C for 45 s, 55 °C for 45 s, 72 °C for 45 s. Following the PCR reaction, the products were subjected to a dissociation analysis to detect the presence of non-specific primer-dimers and a sample of each reaction was analysed by agarose gel electrophoresis in the presence of ethidium bromide staining to verify visually that products of only the expected sizes were present and to confirm the absence of primer-dimers. The resulting real time PCR data were analysed using the comparative C_T_ method [44] and statistical analysis was performed using an analysis of variance (ANOVA).

### Western blot analysis

Salivary glands from female *An. gambiae*, *An. stephensi* and transgenic *An. stephensi* containing transgene As-AAPPSaglin were dissected in cold PBS at 7 days post emergence. Approximately 40 medial and lateral (proximal + distal) glands were separated, and placed separately in 50 μL of Lämmli sample buffer (BioRad, Hercules, CA) with 5% β-mercaptoethanol and homogenized. Equal numbers of medial and lateral lobe equivalents (10 or 25) were loaded into wells and fractionated in a 10% Tris HCL polyacrylamide gel (BioRad) using a broad range Prestained Protein Marker (New England BioLabs, Ipswich, MA) as a molecular weight standard and transferred by electro-blotting to a polyvinylidene fluoride membrane (BioRad). Blots were probed simultaneously with mab2A3 (1:500) and an actin antibody that recognizes all actin isoforms (loading control; rabbit pan-actin antibody; 1:1000; 4968; Cell Signaling Technology, Danvers, MA). Blots were subsequently probed with secondary antibodies specific to mouse (Goat αMouse Cy5, PA45009V) or rabbit (Goat αRabbit Cy3, 28901106V) (GE Healthcare Piscataway, NJ) immunoglobulin G, each diluted at 1:2500. Blots were visualized using a Typhoon Trio™ Variable Mode Imager (GE Healthcare).

## Results

### Spatial and temporal patterns of Saglin expression in *Anopheles gambiae*—protein immunolocalization studies

**Fig. 1:**
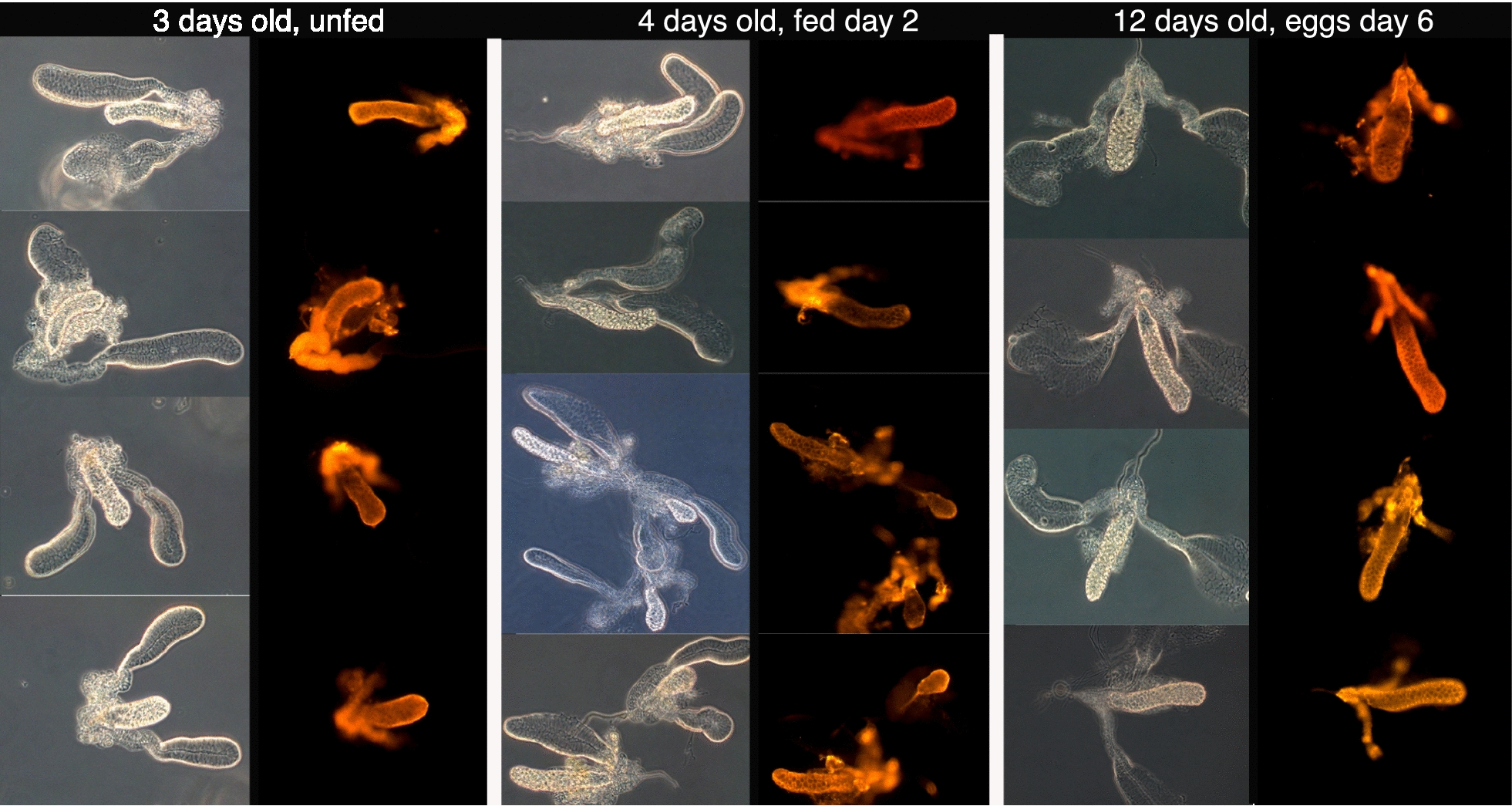
Indirect immunofluorescence analysis of salivary glands from adult female *Anopheles gambiae* of different ages. *Anopheles gambiae* Saglin-specific monoclonal antibody mAb2A3 was used to localize Saglin in salivary glands from females of various ages and physiological states (unfed, fed, pre- and 6 days post-oogenesis). The anti-Saglin antibody mAb2A3 was localized with an Alexafluor 555-labeled goat-anti-mouse secondary antibody and visualized using a fluorescence compound microscope. Salivary glands are shown in pairs of images, one taken using phase contrast optics and the other under fluorescence illumination. Without exception, Saglin was detected in the medial and proximal lateral lobes. No fluorescence was detected in the distal lateral lobes of the salivary glands

Saglin in the salivary glands of female *An. gambiae* displayed consistent temporal and spatial patterns of protein expression. Salivary glands from female *An. gambiae* 3, 4 and 12 days post emergence showed evidence of abundant Saglin only in the medial and proximal lateral lobes (Fig. 1). Saglin protein was undetectable in the distal lateral lobes of female salivary glands under all conditions tested. The physiological state of females with respect to blood feeding and oogenesis had no detectable effect on the spatial pattern of Saglin expression; expression remained confined to the medial and proximal lateral lobes of the salivary glands (Fig. 1).

**Fig. 2:**
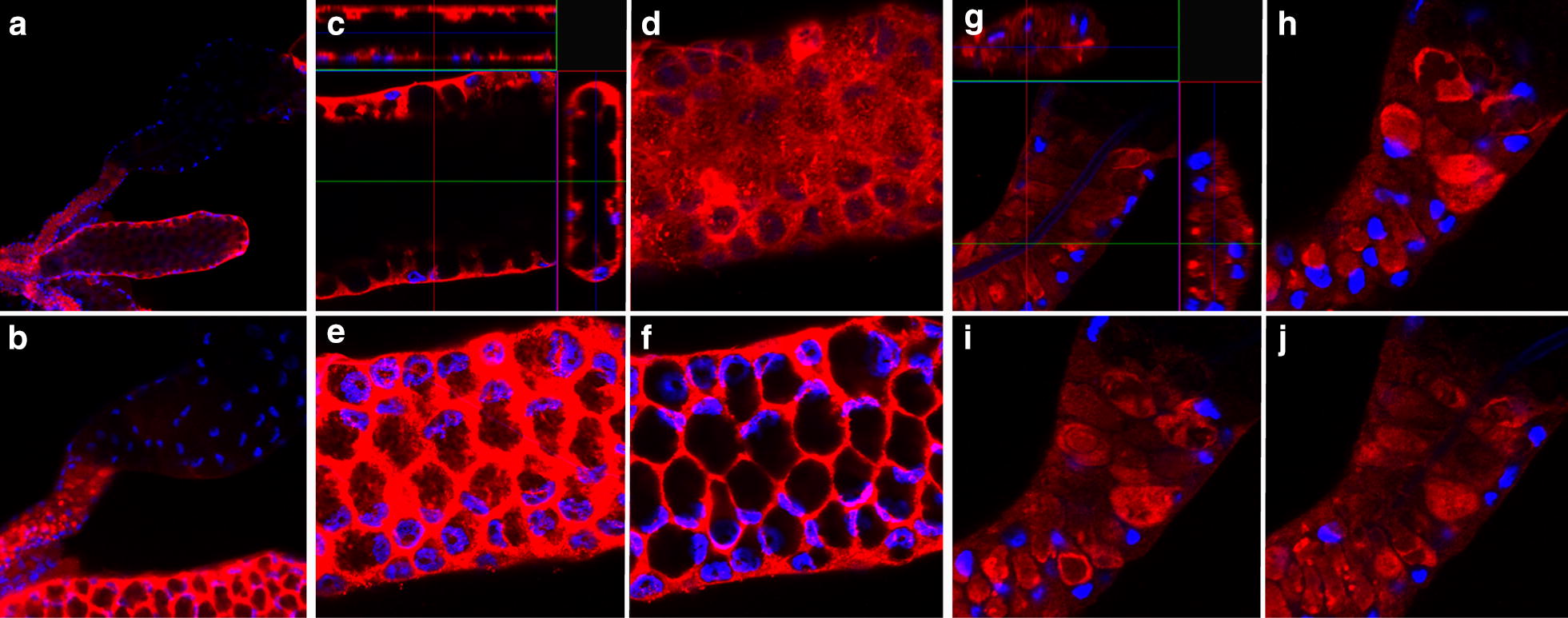
Confocal imaging of immunolocalized Saglin in the salivary glands from adult female *Anopheles gambiae*. **a** A low magnification image (×200) of medial and lateral lobes with evidence of Saglin only in the medial and proximal lateral lobes. **b** The same sample as in panel A but the fluorescence signal associated with Saglin localization in the medial gland was overexposed by adjusting the gain during signal processing. Only under these conditions could a weakly fluorescent signal be detected in the distal lateral lobe. **c**–**f** Selected 1 µm optical frontal sections through parts of the medial gland shown in **a** and **b**. **c** Shows digitally reconstructed sagital- and transverse-sections revealing the presence of Saglin only in basal regions. **d**–**f** Are successive sections beginning at the surface of the gland. The honeycomb appearance is due to the acinar structure of the gland, the presence of large secretory cavities and the basal distribution of Saglin. **g**, **h** Selected 1 µm optical frontal sections through parts of the proximal lateral gland where it joins with the distal lateral region shown in **a** and **b**.** g** shows digitally reconstructed sagittal- and transverse-sections revealing the presence of Saglin throughout the cytoplasm of the cells of this region. Red fluorescence (Alexafluor 555) indicates the presence of Saglin and blue fluorescence (DAPI) indicates DNA. Note that the salivary duct in the proximal lateral region non-specifically binds DAPI and does not indicate the presence of DNA

A confocal microscopy analysis of Saglin distribution in salivary glands from females revealed a number of distinctive features of this protein. Saglin was clearly localized exclusively to the basal surface of the cells of the medial lobe with no reactivity along the lateral or apical cell surfaces or within the secretory cavity and duct (Fig. 2). The pattern of Saglin distribution was notably different in the cells of the proximal lateral lobes of the salivary gland. The proximal cells of the lateral lobes abundantly expressed Saglin that, based on the relative fluorescence intensity, was comparable to that seen in the medial lobe. Saglin expression terminated abruptly at the transition between proximal cells (no secretory cavities) and distal cells (with secretory cavities) (Fig. 1). Saglin was seen uniformly throughout the proximal cells of the lateral lobes (Fig. 2) and was not confined to the basal cell surface as in the cells of the medial gland.

### *Saglin* transcript and protein expression in *Anopheles gambiae* salivary glands

**Fig. 3:**
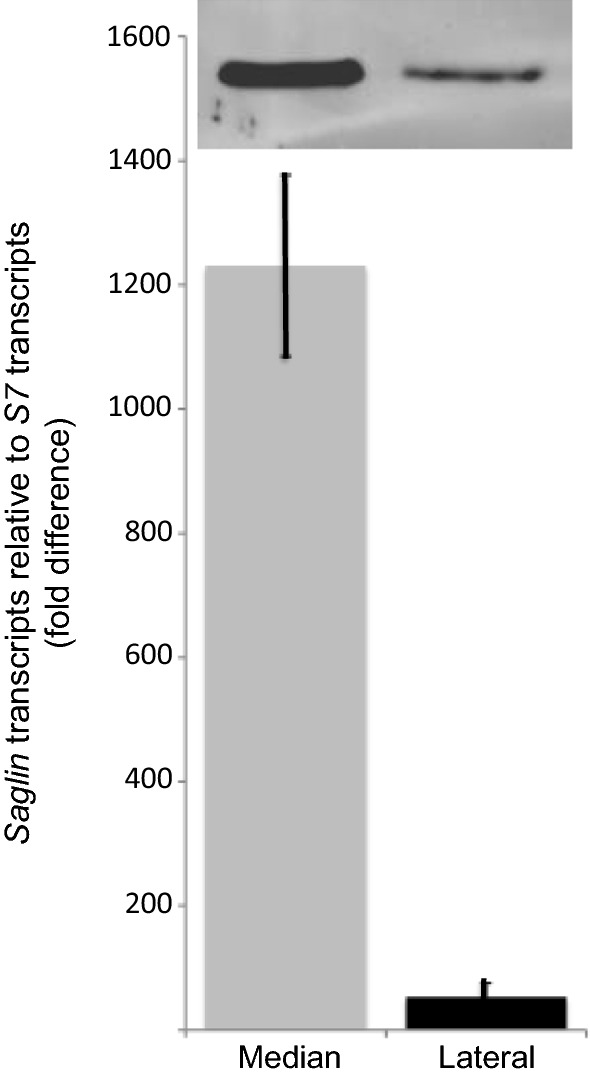
Saglin transcript and protein detection in female *Anopheles gambiae* salivary glands. The transcript levels of *Saglin* in the salivary glands of female *An. gambiae* relative to the transcript levels of the *S7* ribosomal protein gene are shown as the the mean fold difference ± standard deviation. The data show the results of three independent biological replicate experiments (i.e. three independent collections of salivary glands) with transcript levels within each replicate being measured in triplicate. The results of a Western blot showing Saglin protein expression are shown above the histogram

The number of transcripts in the medial lobe of salivary glands from 5 day old adult female *An. gambiae* was over 1000-fold higher than of *S7* transcripts while in the lateral lobes the number of transcripts of *Saglin* were only approximately 50-fold higher than those of *S7* (*F*_*1,4*_=189.881, *P *= 0.00016) (Fig. 3). In these experiments RNA was isolated from the entire lateral lobe of the salivary glands including the morphologically distinct proximal and distal regions. Saglin protein expression, as shown by Western blot, was much higher in the medial lobe of the salivary gland compared to the distal lobes, which was consistent with qRT-PCR and indirect immunofluorescence data.

### Exogenous expression of *Anopheles gambiae* Saglin in the salivary glands of transgenic *Anopheles stephensi*

**Fig. 4:**
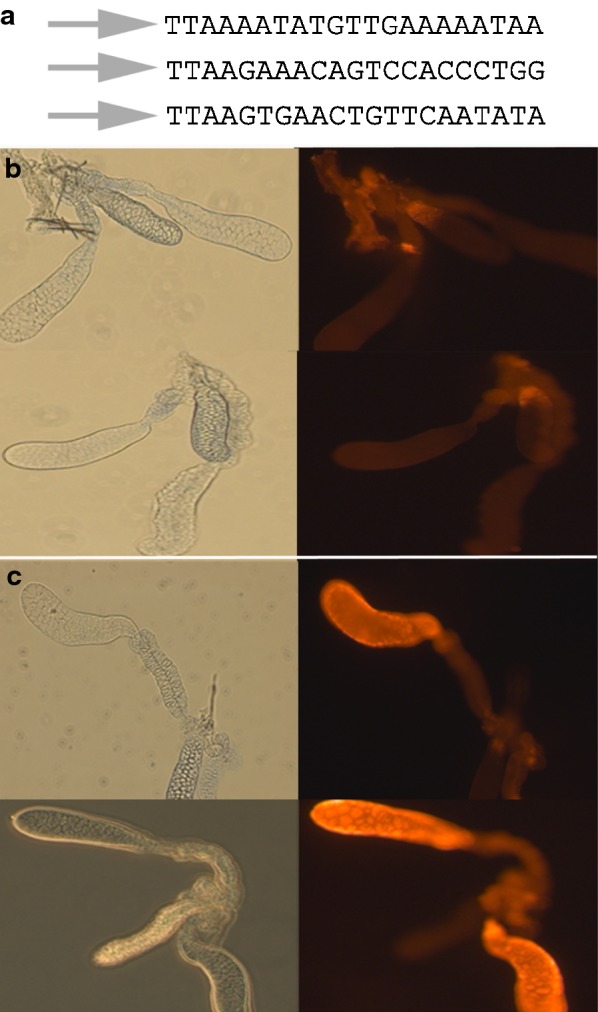
Transgenic *Anopheles stephensi* expressing *Anopheles gambiae Saglin* in the distal lateral lobes of the salivary glands of mosquitoes. **a** DNA sequence flanking the terminal inverted repeats (arrows) of the three *piggyBac* vectors present in the transgenic line used in this study. **b** Phase contrast and fluorescence images of salivary glands from non-transgenic females prepared for indirect immunofluorescence to localize *An. gambiae* Saglin using mAb2A3 as the primary antibody and Alexafluor 555-labeled goat-anti-mouse secondary antibody. No Saglin was detectable. **c** Phase contrast and fluorescence images of salivary glands from transgenic females prepared in parallel with those shown in **b**. Strong fluorescence was detected only in the distal lateral lobes of the salivary glands, as expected based on the known spatial patterns of expression of the *AAPP* promoter from *An. stephensi* that was used to regulate the expression of the *An. gambiae Saglin* transgene

**Fig. 5:**
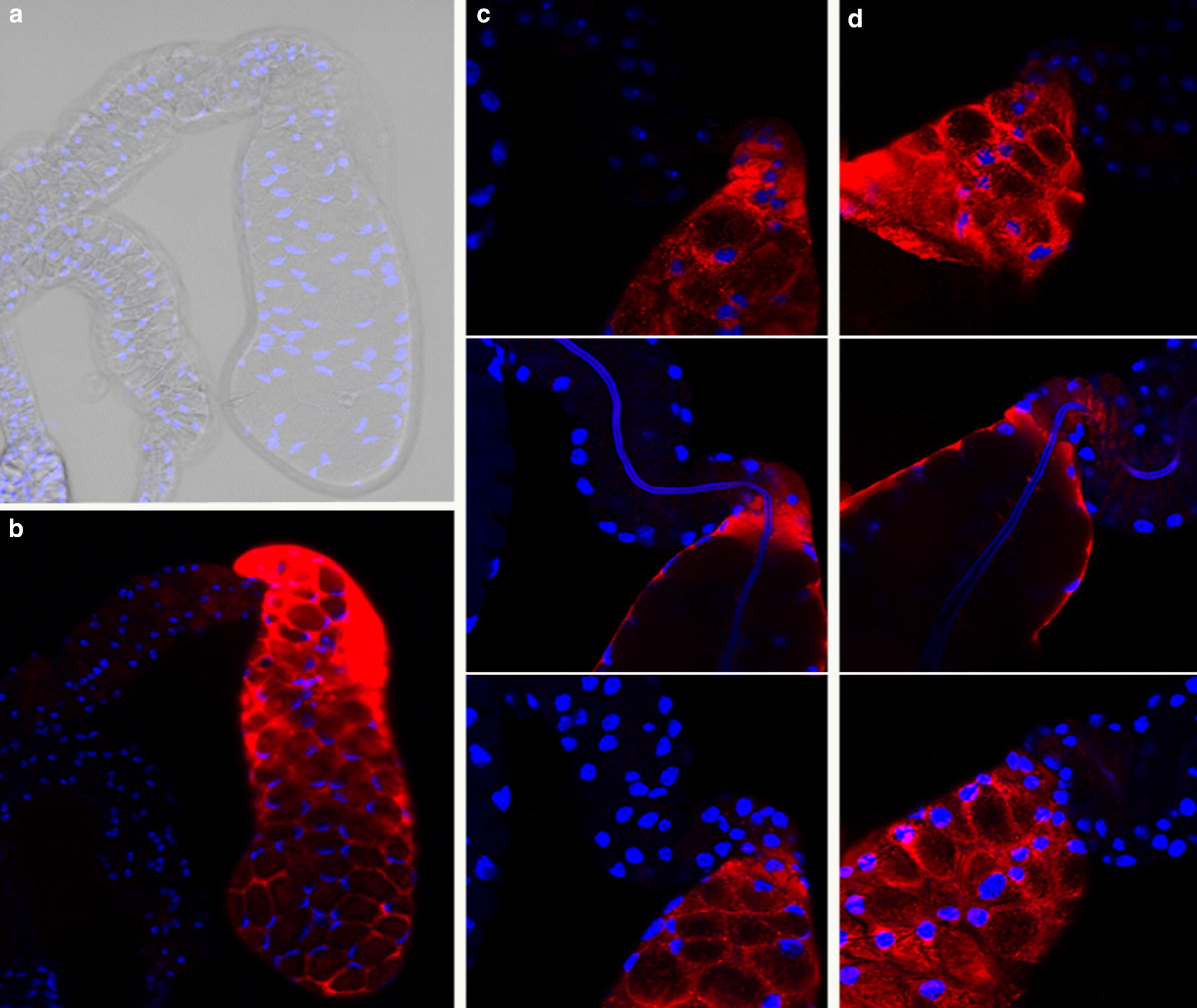
Confocal imaging of immunolocalized Saglin in the salivary glands of transgenic adult female *Anopheles stephensi*. **a**, **b** A low magnification image (×200) showing the proximal regions of two lateral lobes and a single distal lobe. Fluorescence was detected only in the distal region. **c** and **d** High magnification images (×630) of the junction between the proximal and distal regions of two lateral lobes. Each panel consists of three selected optical sections. Fluorescence was only detected in the basal regions of the cells. Red fluorescence (Alexafluor 555) indicates the presence of Saglin and blue fluorescence (DAPI) indicates DNA. Note that the salivary duct in the proximal lateral region non-specifically binds DAPI and does not indicate the presence of DNA

A transgenic line of *An. stephensi* was created that contained three inserts of the gene vector containing a transgene consisting of the Saglin open reading frame under the regulatory control of the promoter from *AAPP*. Inverse PCR was used to isolate and sequence the DNA immediately adjacent to the inverted terminal repeats of the *piggyBac* vector (Fig. 4). Vector integration occurred through typical *piggyBac* transposition as indicated by the presence of TTAA direct repeats adjacent to the terminal inverted repeats of the transposable element (Fig. 4). Strong *DsRed* marker-gene expression was observed in the brain, ventral nerve cord and anal papillae of larvae as is expected based on the known pattern of expression of the *3XP3* promoter in insects. That marker gene expression was observed exclusively in the tissues known to express *3XP3* indicating that this line did not suffer from position effects (Fig. 4). In adult transgenic *An. stephensi*, Saglin protein was detected only in the cells of the distal region of the lateral lobes (Fig. 4). The fluorescence intensities observed in co-processed salivary glands of transgenic *An. stephensi* and *An. gambiae* were visually similar. Saglin was detected exclusively on the basal surfaces of the cells of the distal lateral lobes with no evidence of protein expression along the lateral and apical surfaces or in secretory cavities or ducts (Fig. 5).

### *Plasmodium falciparum* infection of transgenic *Anopheles stephensi*

**Fig. 6:**
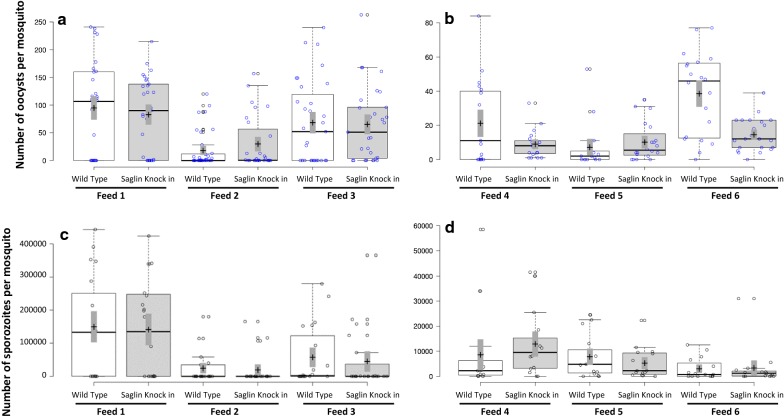
*Plasmodium falciparum* infection characteristics in wild type and Saglin knock in transgenic *Anopheles stephensi*. Results of six experimental feeds in two experiments **a**, **c** and **b**, **d**), each data point showing the number of oocysts (**a**, **b**) and PfSPZ (**c**, **d**) per mosquito. Center lines show the medians; box limits indicate the 25th and 75th percentiles as determined by R software; whiskers extend 1.5 times the interquartile range from the 25th and 75th percentiles; crosses represent sample means; bars indicate 83% confidence intervals of the means; data points are plotted as open circles

In two independent experiments, transgenic and wild type *An. stephensi* were offered blood meals prepared from the same *P. falciparum* gametocyte cultures; within each experiment, three pairs of treatments (wild type versus Saglin knock-in *An. stephensi*) were compared. There were no significant differences in *P. falciparum* oocyst or PfSPZ infection characteristics of heterozygous transgenic and wild type *An. stephensi* (Fig. 6). Geometric mean oocyst intensities did not differ significantly between control and experimental groups in any of the feeds (Mann–Whitney U test between control and transgenic mosquitoes for each paired feed). PfSPZ infections of salivary glands were also compared statistically in the same groups of mosquitoes: neither prevalence rates nor infection intensities differed significantly between the wild type and transgenic *An. stephensi* in any of the three feeds.

## Discussion

*Plasmodium* SPZ released from oocysts invade only the mosquito salivary glands, implying that a specific recognition event is involved. There are few identified candidate proteins with the expected characteristics of a recognition protein and Saglin has been described as such a candidate [30, 32, 33]. The data presented here revise the understanding of the spatial expression pattern of Saglin in the salivary glands as well as its potential role in SPZ-mosquito interactions. Saglin is not detectable in the distal lateral lobes of the salivary glands of female *An. gambiae*, nor does ectopically expressed Saglin in that part of the salivary gland of *An. stephensi* change the prevalence or intensity of PfSPZ infections.

The present observations on the spatial distribution of Saglin within the salivary gland are different from those reported by others. Brennan et al. reported, “…diffuse dispersion throughout the salivary gland”, but this was not apparent from their Fig. 2d used to illustrate their conclusion, which appears to show a medial lobe of the salivary gland [30]. Images were not shown of control salivary glands that demonstrated specificity of the immunofluorescence under the conditions used, nor did they report the age and physiological state of the mosquitoes used in their analysis [30]. Ghosh et al, also reported Saglin localization using immunofluorescence (their Fig. 6c), suggesting there was little Saglin in the proximal lateral region of the salivary glands and approximately equal amounts in both the medial and lateral lobes [32]. The bases for these different observations are not known, but to avoid any ambiguity, the data reported here have been carefully and uniquely controlled. The transgenic *An. stephensi* with the *An. gambiae Saglin* open reading frame under the regulatory control of the promoter from *An. stephensi*’*s AAPP* gene provides a strong positive control for the immunolocalization studies. Because the spatial pattern of transcription of *AAPP* in *An. stephensi* is known and because the immunofluorescence pattern of transgene expression faithfully reproduces the expected pattern, the pattern of Saglin distribution in non-transgenic *An. gambiae* can be assessed confidently knowing that the experimental conditions for localization were optimized. Furthermore, the pattern of *Saglin* expression in the salivary gland was confirmed by both transcript and protein detection studies performed on isolated medial and lateral lobes of salivary glands from *An. gambiae*.

Is Saglin the receptor that enables *Plasmodium* SPZ to invade mosquito salivary glands? Based on the distribution of Saglin in the salivary glands and its inability to enhance infection when over-expressed in the distal lateral lobes of the salivary glands, it would appear that it is not a general receptor for *Plasmodium* parasites although it could play such a role in the medial lobe.

PfSPZ are the active immunogens of PfSPZ vaccines and PfSPZ Challenge [25, 26] and are manufactured in aseptically reared *An. stephensi* mosquitoes. In addition to clarifying the role of Saglin in mosquito-PfSPZ interactions, it was hoped that an *An. stephensi* transgenic line over-expressing Saglin would provide a vehicle for improved PfSPZ intensities and thereby increase the efficiency of PfSPZ manufacturing. Unfortunately, this was not the case but the approach here demonstrates that the generation of robust genetically-modified *An. stephensi* capable of harbouring high intensity PfSPZ infections is indeed possible. Alternative mosquito molecular targets are currently being pursued.
